# Overlapping features of atopic dermatitis and alopecia areata: from pathogenesis to treatment

**DOI:** 10.3389/fimmu.2025.1641918

**Published:** 2025-09-03

**Authors:** Jiayi Cheng, Yugu Jiang, Qianqian Chen, Min Xiao

**Affiliations:** ^1^ Department of Dermatology, Chengdu University of Traditional Chinese Medicine, Chengdu, Sichuan, China; ^2^ Department of Dermatology, The Affiliated Hospital of Chengdu University of Traditional Chinese Medicine, Chengdu, Sichuan, China; ^3^ Guizhou Hospital of Guangdong Provincial Hospital of Traditional Chinese Medicine, Guiyang, Guizhou, China

**Keywords:** alopecia areata, atopic dermatitis, overlap, cytokines, immune dysregulation

## Abstract

Atopic dermatitis (AD) and alopecia areata (AA) have traditionally been regarded as inflammatory dermatoses with independent pathogenic mechanisms, with the former mostly categorized as a type 2 inflammatory disease and the latter as a type 1 inflammatory disease. However, immunologic studies have shown that the immunologic properties of AD and AA do not strictly follow the traditional classification. Both diseases are associated with systemic Th1, Th2, Th17, and Th22 cytokine imbalances, shared genetic susceptibility loci, overlapping immune pathways, and microbiome-mediated modulation of skin pathology. This review systematically investigates the intricate interactions between AD and AA, focusing on shared pathophysiologic mechanisms such as immune network crosstalk, metabolic dysregulation, and microbial influences. Furthermore, it critically evaluates current therapeutic strategies for overlapping disease manifestations, with a detailed analysis of emerging targeted therapies and their implications for clinical practice. By integrating existing evidence and identifying research gaps, this article aims to provide new perspectives on the understanding of the mechanisms of AD-AA interactions and to inform clinical decision-making and future research directions.

## 1 Introduction

Atopic dermatitis (AD) is a chronic, relapsing, multifactorial inflammatory skin disorder characterized by eczematous lesions such as erythema, papules, exudation, xerosis, and pruritus. The Global Burden of Disease Study shows that AD is the most burdensome of the dermatologic diseases and ranks among the top non-fatals (1). Alopecia areata (AA) is a chronic tissue-specific autoimmune disease characterized by non-scarring alopecia affecting approximately 2% of the population (2, 3). Although traditionally viewed as distinct entities, emerging evidence highlights a bidirectional epidemiologic link between AD and AA, underpinned by shared immunopathogenic pathways. The convergence of genetic susceptibility, environmental triggers, epidermal barrier dysfunction, microbiome dysbiosis, and immune dysregulation collectively drive the pathogenesis of both conditions (4–6). Notably, atopic predisposition—particularly a history of AD—is significantly overrepresented in AA cohorts and serves as a key risk factor for AA development (7). Conversely, AD patients exhibit a markedly elevated risk of AA onset (8). However, the existing literature suffers from an insufficient understanding of the comorbidity mechanism and a lack of consistency in treatment protocols, etc. This review explains this association from the perspectives of the interactions between AD and AA, clinical characteristics, and treatment strategies, with the aim of providing references for an in-depth understanding of the mechanisms of the comorbidity between the two and for the development of effective interventions.

## 2 Epidemiology

In a retrospective study of 51,561 patients with AA, Kridin et al. identified a robust bidirectional association between AA and AD (9), showing that AD and AA are most frequently comorbid compared to other atopic diseases, and that AA patients with comorbid AD presented an earlier disease onset and a higher prevalence of female patients. In addition, a key observation was that the risk of AA in atopic disease patients correlated with the type of comorbid atopy. However, it is worth noting that the database did not include information on the severity of AA versus AD, so it was not possible to delve into the specific association between disease severity and increased risk. A Korean retrospective study of 871 patients with early-onset (prepubertal) AA further supports these findings (10), highlighting AD as the most prevalent comorbidity in this population. Similarly, Conic et al. observed a high co-prevalence of AD of 17.4% in 3,510 AA patients under 18 years of age by analyzing data from 26 major healthcare networks in the United States (covering more than 360 hospitals) (11), suggesting that this bi-directional correlation is also present in pre-pubertal patients. Notably, a Taiwanese cohort study involving 12,022 AA patients and 40,307 AD patients (8), not only reiterated the bidirectional increased risk between AD and AA, but also demonstrated that AD patients carrying *Filaggrin* gene (*FLG)* mutations exhibited exacerbated AA manifestations compared to those without genetic predisposition, further strengthening the further reinforcing the complex and multidimensional association between AD and AA.

## 3 Complex immune networks

**Figure 1:**
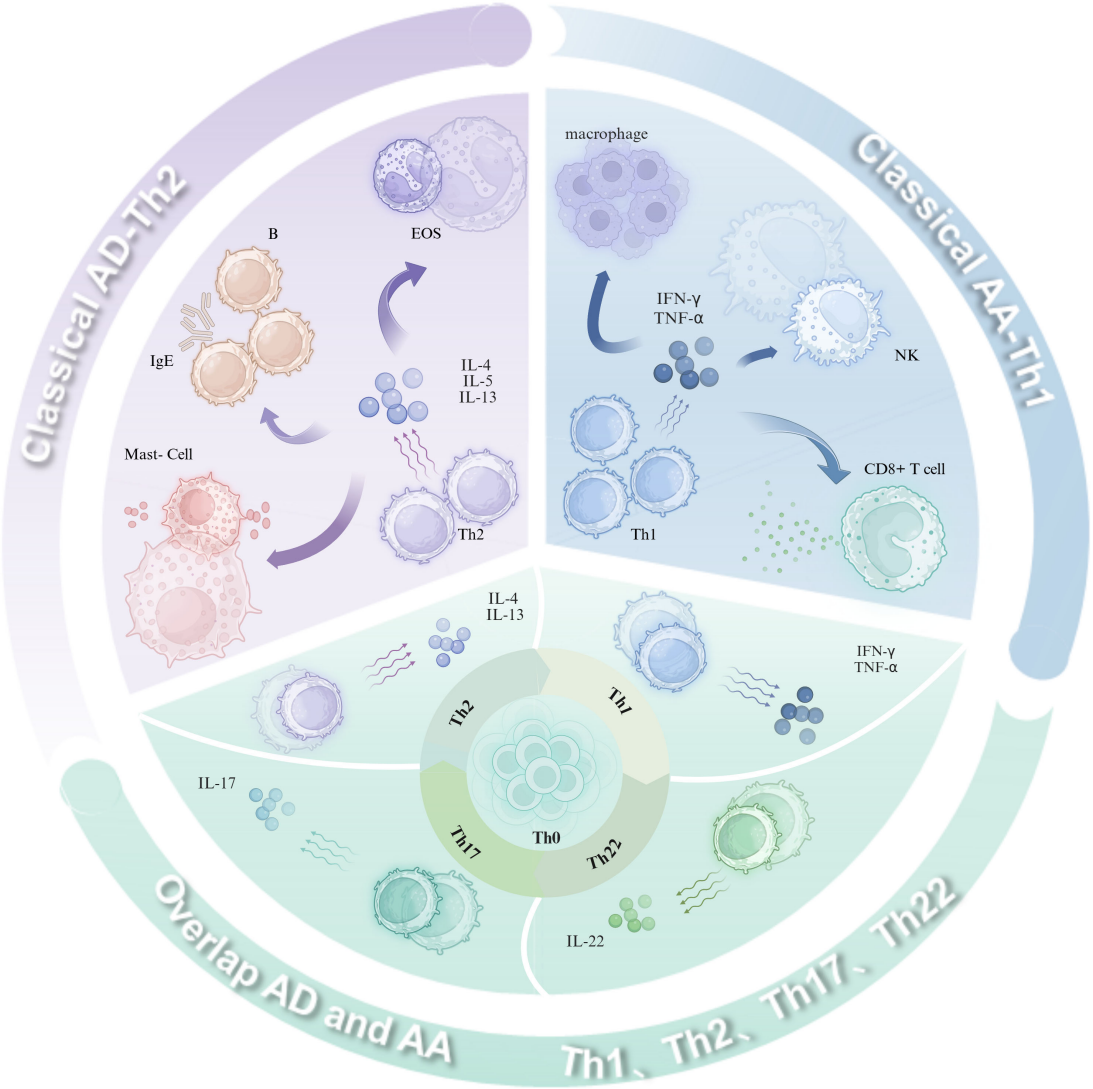
Immunopathogenesis of AD, AA, and their overlap. Classical AD is characterized by a Th2-predominant immune-inflammatory response, where cytokines such as IL-4, IL-13, and IL-31 stimulate B-cell production of IgE, promote mast cell degranulation, and recruit Eos. In contrast, classical AA is driven by a Th1-dominant immune-inflammatory response, with cytokines like TNF-α and IFN-γ activating macrophages, CD8^+^ T cells, and inducing NK cell activation. In overlapping AD and AA, Th1, Th2, Th17, and Th22 cells collectively contribute to disease progression through the secretion of IL-4, IL-13, IL-17, IL-22, TNF-α, and IFN-γ. (Figure created with BioRender.com). AD, Atopic dermatitis; AA, Alopecia areata; Th, T helper cells; IL, Interleukin; IFN, Interferon; TNF, Tumor necrosis factor; Eos, Eosinophils; NK, Natural killer cells; CD8^+^ T cells, CD8-positive cytotoxic T cells.

AD is mainly mediated by Th2-driven immune responses, and key cytokines such as interleukin (IL)-4 and IL-13 play a central role in skin barrier disruption, promoting immunoglobulin (Ig)E production, and modulating the inflammatory process (12). Based on the concentration of IgE and the status of the skin barrier, AD is further classified into two distinct subtypes: exogenous and endogenous. Specifically, exogenous AD is characterized by high serum total IgE levels, significantly increased expression of Th2-type cytokines, and impairment of skin barrier function. In contrast, endogenous AD exhibits normal serum total IgE concentrations, low expression levels of Th2-type cytokines, and relatively intact skin barrier function (13). Further research indicates that in the presence of AA, AA is more inclined to exhibit a skewed Th1-type immune response when combined with endogenous AD. In contrast, it is more likely to present a skewed Th2-type immune response when combined with exogenous AD (14). This immune dynamic manifests temporally in AD progression: Th2 predominance characterizes the acute phase, while Th1 dominance emerges during chronic stages (15, 16). Critically, cross-regulation between Th1-derived cytokines (e.g., IFN-γ) and Th2-associated IL-4/IL-13 orchestrates the evolving immune microenvironment (17). This mechanism of immune switching from Th2 to Th1 may contribute to the chronic progression of AD, exacerbating the complexity and intractability of the disease. AA pathogenesis is predominantly mediated by Th1 cell-derived interferon-gamma (IFN-γ) and tumor necrosis factor-alpha (TNF-α), which elicit immune responses to environmental stressors (e.g., psychological stress, viral infection, trauma), resulting in follicular immune dyshomeostasis and compromised hair growth (18). Interestingly, the pattern of immune response in AA patients is equally complex and diverse, also involving Th2 cytokines (IL-4, IL-5, IL-10), IgE, and eosinophils (3, 19). Collectively, these findings challenge the traditional Th1/Th2 dichotomy, revealing a continuum of immune activation in AD and AA. The concurrent activation of Th1, Th2, and Th17/Th22 axes—interconnected through comorbid crosstalk—drives disease pathogenesis and progression (20, 21). These observations indicate that immune responses in AD and AA form a dynamic continuum rather than distinct binary classifications, with Th1, Th2, and Th17/Th22 pathways interwoven under comorbid states. This interplay jointly influences disease pathogenesis and progression, while the simultaneous activation of multiple T-helper (Th) cell subsets underscores the necessity to understand immune equilibrium in diverse dermatoses for therapeutic optimization. Immunopathogenesis of AD, AA, and their overlap (**Figure 1**).

### 3.1 Th1-type immunity

The core physiological function of IFN-γ, as a central cytokine of the Th1-type immune response, focuses on recognizing and defending against intracellularly parasitized viruses and malignant cells (22, 23) and has been established as a key mediator in the pathogenesis of AA. In the serum and lesion sites of AA patients, the upregulation of IFN-γ and TNF expression was positively correlated with disease severity and duration, especially in total/purple baldness and active AA (21, 24–26), which effectively promotes immune cell recruitment and exacerbates immune responses mediated by Th1 and natural killer (NK) cells by inducing the enhancement of chemokine expression (27), a cascade of biological events that severely disrupts the natural cycle of hair growth and the normal function of the hair follicle. Emerging evidence highlights IFN-γ’s critical involvement in AD. Stratification of AD patients according to IFNG expression levels and IFN-γ-secreting T cell capacity reveals two distinct subgroups: IFN-γ-high AD (exhibiting predominant endogenous AD characteristics) and IFN-γ-low AD (manifesting classical exogenous AD features) (28), which is consistent with previous studies (29, 30). Pathway analysis revealed that significant enrichment of gene sets associated with innate immunity, lymphocyte activation, inflammatory signaling, and immune system processes in the IFN-γ-high AD subgroup. IFN-γ and TNF-α bind to keratinocytes, induce apoptosis, and create a pro-inflammatory environment, leading to inflammatory skin lesions in AD. Meanwhile, IFN-γ also disrupts the skin barrier homeostasis by down-regulating the expression of *FLG* and mitogenin-1 and ceramide synthesis (31, 32). This barrier dysfunction facilitates inflammatory mediator release, creating a self-perpetuating cycle of immune activation and tissue damage.

### 3.2 Th2-type immunity

In both AD and AA, a Th2-skewed immune response is observed (33), characterized by elevated levels of type 2 inflammatory cytokines, predominantly IL-4 and IL-13. IL-4/IL-13 can combine with type 1 cytokines (e.g., IFN-γ) to upregulate eosinophil-activating chemokines, including eotaxin-1 (CCL11) and eotaxin-3 (CCL26). These chemokines demonstrate significantly elevated expression levels in lesional AD skin (34), driving the recruitment of T lymphocytes, eosinophils, and basophils to the skin lesions. Furthermore, IL-4/IL-13 signaling downregulates key epidermal barrier molecules including *FLG* and involucrin, compromising tight junction integrity in the skin, whereas *FLG* gene mutations constitute the primary genetic determinant of epidermal barrier dysfunction (35), and also reduces ceramide synthesis and impairs corneal cohesion (20, 36, 37). The Th2 cytokine interleukin-4 disrupts normal stratum corneum cohesion in mice, providing implications for AD pathogenesis. Th2 cells are also shown to have a skewed cytokine homeostasis in patients with AA state. Specifically, IgE levels are significantly elevated in AA patients and this elevation is not dependent on the atopic state (38). At the same time, Th2-associated cytokines (IL-4, IL-13) also showed increased levels (39), and these cytokines may further promote T-cell infiltration into the skin and hair follicles, thereby triggering inflammatory responses and follicular damage (33, 40), and in the group of patients with AA combined with AD, the infiltration of Th2 cells around the diseased follicles was more prevalent. prevalent (14), and there was a significant correlation with IL-13 levels (41). In addition, it was further noted that enhanced activation of both skin-homing and circulating Th2 cells in AA patients versus healthy controls, with activation intensity directly correlating with clinical severity scores. Conversely, IFN-γ expression demonstrates stronger association with chronic disease progression (42).This observation aligns with the established immune trajectory of AD: The acute phase is predominantly characterized by a Th2 response, where IL-4/IL-13 signaling via the STAT6 (Signal Transducer and Activator of Transcription 6) pathway suppresses IFN-γ signaling. In contrast, the chronic phase exhibits significantly upregulated IFN-γ (43). This elevated IFN-γ can stimulate keratinocytes to produce CXCL10, which subsequently recruits cytotoxic CD8^+^ T cells to infiltrate hair follicles, ultimately triggering the autoimmune attack characteristic of AA. Thus, the Th2-to-Th1 immune shift occurring during the AD chronicity phase serves as a pivotal link connecting prolonged AD to AA pathogenesis.

### 3.3 Th17/Th22-type immunity

Imbalances in the immune axis play a central role in the development of AD. Specifically, the increase in Th1/IFN-γ-associated products may not only signal a pro-inflammatory activation state of Th17/Th22 cells, but may also serve as a mechanism of counter-regulation of Th2 and Th17 activation (44, 45). Gittler et al. emphasized the strong association between the enhancement of this immune axis and the increase of immune activation in the progression of AD to the chronic phase, and this immune imbalance is already evident in the acute lesion stage (16), especially with a significant increase in Th22, Th2 and Th1-related products. Critically, IL-17 and IL-22, as key pro-inflammatory cytokines for Th17 and Th22 cells, exert pivotal roles in AD pathogenesis through distinct mechanisms. IL-22 upregulates gastrin-releasing peptide receptor expression in keratinocytes of the skin (46), which is key in mediating both non-histaminergic and pathologic itch (47, 48), thus affecting the itch symptoms in AD itch symptoms in patients. Simultaneously, Th17 cell activation in AD facilitates the generation of pro-inflammatory cytokines, which consequently might intensify inflammation and lead to alopecia (49, 50). In AA, perifollicular dermal infiltration by Th17 cells coincides with significantly elevated serum IL-17 levels compared to healthy controls, which is strongly correlated with the severity of AA (51). In addition, higher serum IL-17A levels in young AA patients may be associated with their increased susceptibility to psychological stress (52), and this susceptibility may stem from chronic stress-induced conversion of lymphocytes to Th17 responses (53). Therefore, adolescent-onset AA patients may portend a poor prognosis due to high IL-17A levels compared to those with advanced age onse (54). On the other hand, Atwa et al. observed that AA patients displayed elevated serum IL-22 levels compared to healthy controls, with a positive correlation with AA and depression duration (51, 55). These findings further support the critical role of immune imbalance in the chronic pathology of AD and AA.

### 3.4 JAK-STAT signaling pathway

The JAK (Janus Kinase) -STAT signaling pathway, as a core intracellular transduction mechanism for cytokines, critically regulating organism development, maintaining homeostasis, promoting cell proliferation and mediating immune responses. In the pathological process of AD, this pathway significantly affects keratinocyte function through JAK/STAT-dependent signaling of IL-4 and IL-13 (56). This dysregulation is demonstrated by suppressed *FLG* and endocannabinoid production, subsequently compromising skin barrier integrity (57, 58). Notably, STAT6 protein plays a central regulatory role in this signaling pathway, which not only upregulates the expression of the Th2-specific transcription factor GATA3 and regulates T cell proliferation and Th2 cell differentiation, but also promotes the conversion of immunoglobulin classes to IgE and IgG1 in B cells (59), which correlates with the fact that multiple STAT6 polymorphisms are associated with high levels of IgE and an increased AD susceptibility is closely associated with increased susceptibility (60). In addition, the STAT3 component of the JAK-STAT signaling pathway plays an amplifier role in the development of chronic itch (61), and its activation is directly associated with sustained itch signaling. Genome-wide association studies (GWAS) in AA patients have identified JAK-STAT pathway components—including STAT5A/B, STAT3, JAK1, and JAK3—as critical regulators of follicular cycling (62, 63). The up-regulated expression of these genes during the regression and resting phases, as well as their down-regulation during the early anagen phase, implies that JAK-STAT signaling may inhibit hair re-entry into the anagen phase (64). In contrast, JAK inhibitors have indeed demonstrated a significant ability to promote hair regrowth in clinical studies (65), as evidenced by elevated post-treatment hair keratin content, a reduction in perifollicular T-lymphocyte infiltration, and a significant down-regulation of inflammatory markers in the gene expression profile (66, 67).

### 3.5 OX40-OX40L signaling pathway

OX40 and its ligand OX40L are both key members of the TNF superfamily. OX40 is primarily expressed on enhanced effector T cells (including Th1, Th2, Th17, and Th22 subpopulations) and regulatory T cells (Treg), while OX40L is primarily expressed on activated antigen-presenting cells (68). Ilves et al. found that OX40^+^ T cells were increased by 10-fold in the skin of AD patients compared to non-lesional skin (69). In the pathophysiology of AD, the OX40-OX40L signaling pathway plays a crucial role, promoting Th2 cell differentiation, These activated Th2 cells not only express OX40 but also release cytokines, further exacerbating damage to the epidermal barrier function. Preclinical studies in skin inflammation and asthma models further support that the OX40-OX40L signaling interaction is critical for the efficiency of regulatory responses in memory Th2 cells (70, 71). Additionally, this signaling pathway promotes the recruitment and proliferation of Th1, Th17, and Th22 cell subsets, which mediate keratinocyte proliferation, epidermal thickening, and T cell recruitment by upregulating the production of cytokines such as IFN-γ, IL-17, and IL-22 (72, 73). A study on mechanistic biomarkers demonstrated that blocking OX40 signaling not only regulates Th2 characteristics but also simultaneously suppresses Th1 and Th17/Th22-related immune activation (74). However, OX40 and OX40L are not significantly correlated with the clinical severity of AD. Additionally, Xiao et al. found that OX40 signaling significantly inhibits IL-17A production and Th17 differentiation in an experimental autoimmune encephalomyelitis model (75). The role of the OX40L/OX40 pathway in influencing Th cell fate remains to be further elucidated. Nevertheless, the activation of the OX40L/OX40 pathway, which promotes abnormal infiltration of effector T cells and cytokine release, may still be a key trigger for the disruption of follicular immune privilege. It is well known that mast cells (MCs) play a key role in various allergic diseases, including AD. Overactivation of the OX40L/OX40 pathway promotes Th2 cell proliferation, and the IL-4 secreted by these cells can induce B cells to differentiate into IgE-secreting cells. IgE binds to the high-affinity IgE receptor (FcϵRI) on MCs, leading to the release of histamine and IL-6, among other chemokines, which further recruit Th2 cells and eosinophils, exacerbating the immune inflammatory response (76). Additionally, mast cells upregulate OX40L expression due to IgE-FcϵRI cross-linking (77). Recent studies have further demonstrated that MCs are also involved in the pathogenesis of AA (78, 79). Specifically, compared with healthy control skin, the density of MCs in the dermis around lesions and hair follicles in AA patients is significantly increased. By releasing inflammatory mediators such as TNF-α and IL-6, they exacerbate autoimmune reactions, a phenomenon also observed in HF mesenchyme. This may indicate that Th2 cells act as an important bridge between AD and AA through the OX40L/OX40 pathway.

**Figure 2:**
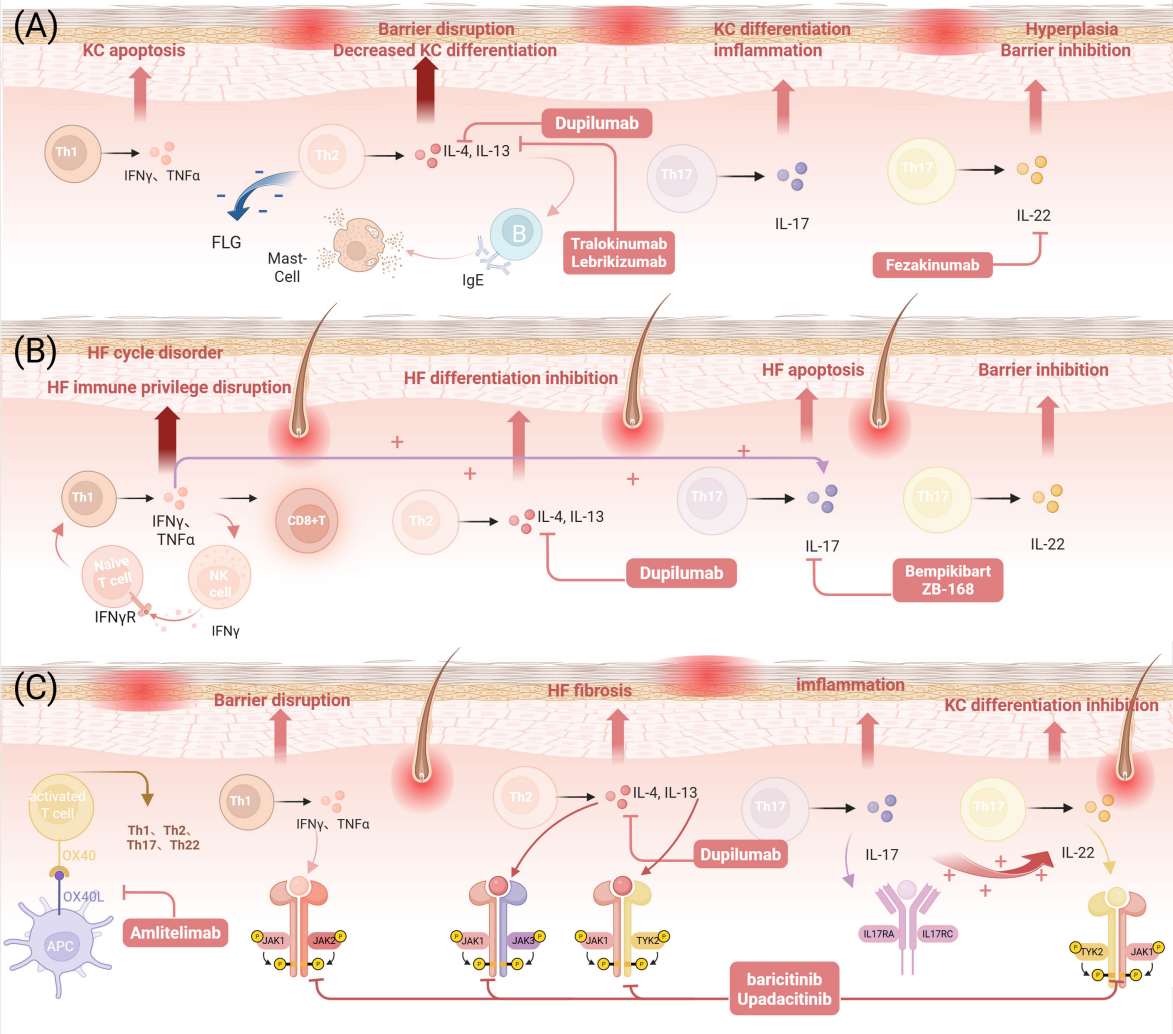
AD, AA, and overlapping immune responses and therapeutic targets. **(A)** The role of Th1/Th2/Th17/Th22 immunity in AD and therapeutic targets. **(B)** The role of Th1/Th2/Th17/Th22 immune responses in AA and therapeutic targets. **(C)** The role of Th1/Th2/Th17/Th22 immune responses, the JAK-STAT signaling pathway, and the OX40-OX40L signaling pathway in AD and AA and therapeutic targets. (Figure created with BioRender.com). AD, Atopic dermatitis; AA, Alopecia areata; Th, T helper cells; IL, Interleukin; IFN, Interferon; TNF, Tumor necrosis factor; KC, Keratinocyte;CD8^+^ T cells, CD8-positive cytotoxic T cells; NK, Natural killer cells; HF, Hair Follicle; APC, Antigen-Presenting Cell; TYK2, Tyrosine Kinase 2; FLG, Filaggrin gene; IgE, Immunoglobulin E; OX40, Tumor Necrosis Factor Receptor Superfamily Member 4; OX40L, Tumor Necrosis Factor Superfamily Member 4 Ligand; JAK, Janus kinase; STAT, Signal Transducer and Activator of Transcription.

Meanwhile, Tregs also occupy a central position in the pathogenesis of AA (80), while OX40L on the MC surface can effectively weaken the immunosuppressive function of Tregs by interacting with OX40 on Tregs. This interaction leads to CD8^+^ T-cell hyperactivation, characterized by excessive IFN-γ and TNF-α production, alongside inflammatory cell recruitment (81, 82), while promoting the production of IgE by B cells as well as the release of Th2 cytokines (77), an interaction that disrupts the homeostasis between Tregs and CD8^+^ T cells and exacerbates the breakdown of immune privilege in AA hair follicles. Recent experimental studies have provided new evidence. Kim et al. found in a large-scale study of moderate-to-severe AA patients that the OX40L/OX40 axis was significantly upregulated in both the follicular surroundings and the circulatory system of patients, and this phenomenon was unrelated to atopic background. Additionally, the study observed an increase in OX40L^+^ antigen-presenting cells (APC) in the circulation, and non-skin-homing Tregs exhibited high OX40 expression, suggesting that at least part of Treg dysfunction may originate in the peripheral blood circulation prior to their migration to the skin (83). Therefore, the OX40/OX40L axis plays a key role in regulating inflammatory responses by modulating the interaction between Tregs and MCs. Abnormal activation of the OX40/OX40L pathway is not only a core driver of chronic inflammation in AD but also a critical link in the susceptibility of AD patients to AA. In AD, the sustained activation of this pathway sensitizes effector T cells and impairs Treg function, leading to an immune imbalance that directly disrupts the microenvironment necessary for maintaining follicular health. Thus, the activation of the OX40/OX40L pathway in AD is an important initiating factor in triggering the autoimmune process of AA. The OX40/OX40L axis, as a potential novel therapeutic target, may offer broad therapeutic benefits for patients with AA and AD comorbidity (**Figure 2**).

## 4 Genetic factors

The contribution of genetic factors to elucidating the pathogenesis of AD and AA has received much attention in recent years. This surge in investigation has been most pronounced in the field of AD research, with genetics being a key word in up to 11% of AD studies over the past decade (84). Similarly, positive family history, a direct reflection of genetic influence, significantly elevates the risk of developing AA and AD. Specifically, up to 48% of people with AA cases exhibit disease manifestation in a relative (85), and children with a history of atopic disease in both parents are five times more likely to develop early-onset AD and persistent phenotypes (86). GWAS studies have revealed significant associations between susceptibility loci in AA and AD, particularly in genomic regions encoding epidermal structural proteins (e.g., FLG) and immune-related genes involved in innate and adaptive immunity (87–89). Epidermal barrier dysfunction caused by *FLG* mutations is considered a major genetic factor for AD, not only increasing the risk of AD, but also strongly associated with early-onset disease and severe clinical phenotypes (90). Notably, *FLG* mutations also elevated the risk of developing AA and exacerbate disease severity in patients with a history of AD (91). The underlying mechanism may involve FLG mutation-induced stratum corneum dysfunction, which compromises the skin barrier in AD. This barrier defect can subsequently disrupt the hair follicle’s immune privilege microenvironment, facilitating the penetration of environmental antigens. The ensuing activation of local immune responses may ultimately trigger AA. Concurrently, chronic inflammation in AA can spread to the skin, inhibiting keratinocyte proliferation and downregulating FLG expression, thereby further aggravating AD-associated barrier dysfunction and establishing a vicious cycle. These findings underscore the pivotal role of FLG mutations as a shared genetic underpinning in mediating the complex comorbid relationship between AD and AA. In addition, another GWAS showed multiple IL-4 promoter polymorphisms in AD patients, suggesting that abnormal IL-4 production is associated with AD susceptibility (92, 93). Similarly, intronic tandem repeat polymorphisms in IL-4 (specifically within intron 3) were identified as AA risk alleles in a Turkish cohort (94). On the other hand, IL-13 gene polymorphisms are linked to both the allergic phenotype of AD and AA susceptibility, with this Th2 cytokine serving as a shared genetic risk factor (93, 95). Meanwhile, IL-13 and KIAA0350/CLEC16A loci—previously associated with autoimmune diseases—mediate genetic overlap between AA and atopy (e.g., allergic rhinitis, asthma) (96), thereby reinforcing the shared etiological framework linking AA, AD, and atopic disorders.

## 5 Microbiome

Emerging evidence from expanding human microbiome studies has established the central involvement of both gut and cutaneous microbiota in the pathogenesis of AD and AA (97, 98). The strong association between gut flora imbalance and AA is partly attributed to the fact that they share a genetic basis capable of inducing a Th1 response and promoting the production of IFN-γ. As a principal immunomodulatory mediator, IFN-γ exerts its biological effects primarily through JAK-STAT pathway activation (99), driving dysregulated follicular keratinocyte proliferation that culminates in hair cycle disruption. Disruption of the gut microbiota not only affects local intestinal homeostasis but may also compromise the integrity of the intestinal epithelial barrier, leading to “leaky gut syndrome”(LGS). This immune homeostasis disruption triggered by microbiota imbalance is considered one of the potential mechanisms exacerbating AA: Rafik et al.’s case-control study showed a positive correlation between intestinal permeability biomarkers and AA severity (100); However, Hacınecipoğlu et al. reached the opposite conclusion, finding no significant association between LGS and AA (101), so the direct causal relationship between increased intestinal permeability and AA still needs further verification. After intestinal barrier disruption, bacterial metabolites can spread through the bloodstream, thereby regulating systemic immune responses and affecting the function of distant organs, including the skin, which forms the core of the “gut-skin axis” theory (102). Specifically, LGS leads to the leakage of intestinal bacteria and their products, which interact with skin receptors to induce Th2-type immune responses, exacerbating skin inflammation in AD (49), and may even trigger systemic pathological processes, including autoimmune diseases (103).

Although the skin microbiota dysbiosis characteristics of AD and AA differ, AD is primarily characterized by reduced skin microbiota diversity and increased *Staphylococcus aureus* abundance (104), while AA is primarily characterized by increased *Propionibacterium acnes* abundance, with no significant changes in the relative abundance of Staphylococcus aureus (105). However, skin microbiome imbalance may weaken the immune privilege state of hair follicles, and the skin barrier dysfunction caused by AD may promote bacterial antigen invasion into hair follicle structures, jointly exacerbating the pathological progression of AA (106). When gut microbiota ferment undigested substrates such as dietary fiber, they produce a class of metabolites known as short-chain fatty acids (SCFA), whose main components are butyrate, propionate, and acetate. These substances exert anti-inflammatory effects through various mechanisms, including maintaining the integrity of the mucus layer and epithelial cells (107). Studies have shown that SCFAs can regulate immune cell activity by activating G protein-coupled receptors, not only inhibiting the release of pro-inflammatory cytokines but also promoting the differentiation and function of Treg cells (108). Treg cells induce and maintain the body’s tolerance to self-antigens by secreting TGF-β and IL-10, playing a key role in preventing autoimmune reactions and maintaining immune homeostasis (109).

**Figure 3:**
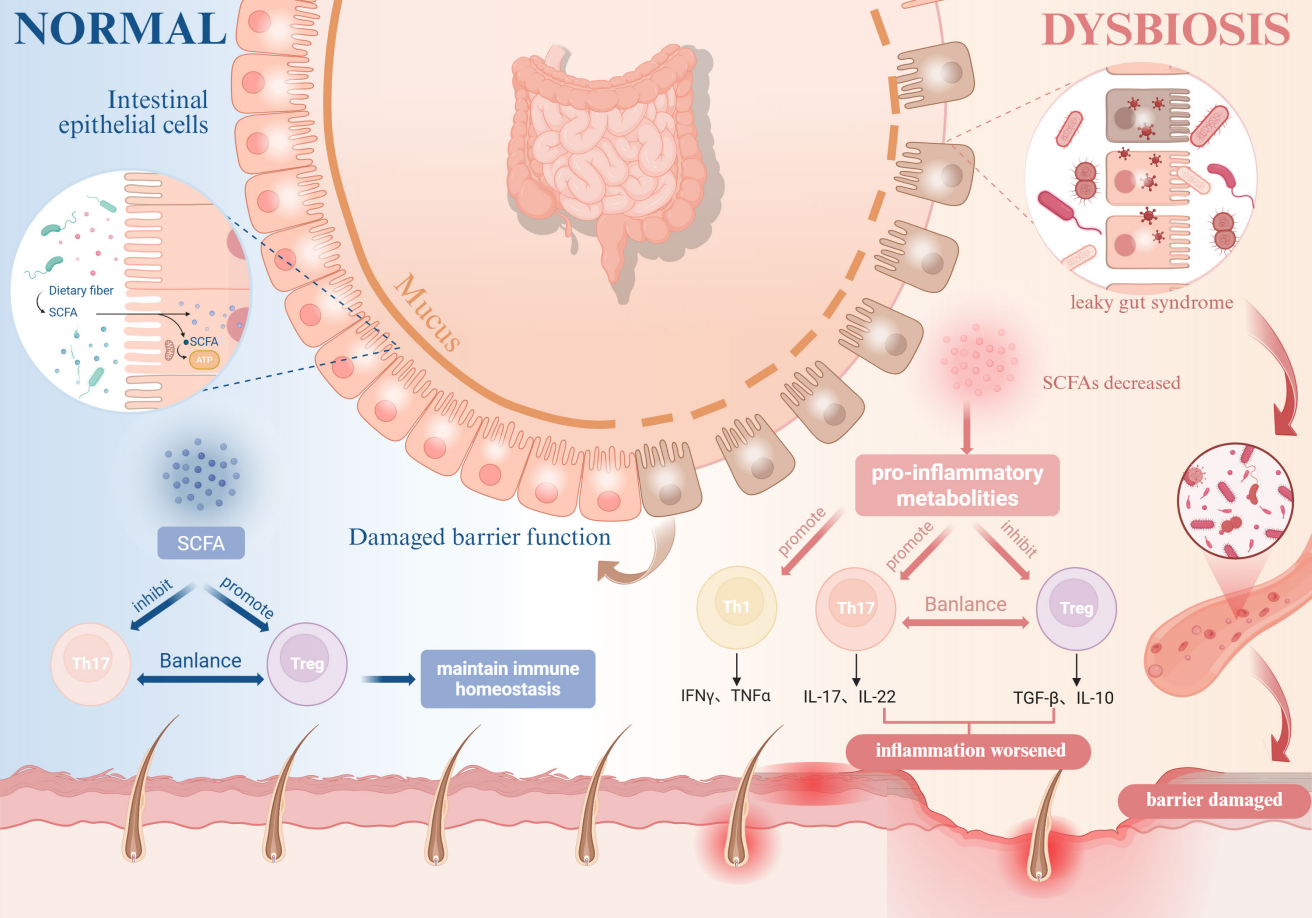
Impact of gut microbiota dysbiosis on AD and AA. Gut microbiota dysbiosis impairs intestinal epithelial barrier integrity, leading to “leaky gut syndrome” and reduced SCFA production. This disruption shifts the balance between pro-inflammatory CD4^+^IL-17^+^ T cells and anti-inflammatory regulatory Tregs, suppresses Tregs functionality and their secretion of anti-inflammatory cytokines (e.g., IL-10), and stimulates the release of pro-inflammatory cytokines such as TNF-α and IFN-γ, thereby exacerbating the pathogenesis of both AD and AA. (Figure created with BioRender.com). AD, Atopic dermatitis; AA, Alopecia areata; SCFA, Short-chain fatty acids; Tregs, Regulatory T cells; IL, Interleukin; TNF-α, Tumor necrosis factor-alpha; IFN-γ, Interferon-gamma.

Conversely, gut microbiota dysbiosis leads to a significant reduction in SCFA production, which may disrupt the balance between pro-inflammatory CD4^+^IL-17^+^ T cells and anti-inflammatory CD4^+^ FOXP3^+^ Treg cells in the gut, promoting abnormal differentiation of Th17 cells and the release of pro-inflammatory factors such as IL-17 and IL-22, thereby inducing tissue inflammation (110), potentially contributing to the onset of AD and AA. Han et al. also confirmed that AA patients exhibit significantly elevated Th17 cell counts and reduced Treg cell counts (111). Therefore, the reduction in SCFAs caused by gut microbiota dysbiosis may be a potential underlying cause of intestinal barrier damage, increased permeability, and exacerbated inflammation in AD patients. While no microbiome-targeted therapies have achieved clinical translation to date, emerging microbiota-modulating strategies show therapeutic promise for inflammatory autoimmune disorders. Importantly, the pathophysiological interplay between AD and AA remains underexplored, necessitating systematic multi-omics investigations to delineate shared microbial etiologies and identify novel therapeutic targets (**Figure 3**).

## 6 Targeted therapy

### 6.1 IL-4/IL-13 inhibitors

Tralokinumab, the first-in-class monoclonal antibody targeting IL-13, was developed for AD. The drug modulates the expression levels of key AD biomarkers in the skin, restoring them to a near-nondiseased state, and effectively reduces the levels of systemic markers of Type 2 inflammation. In addition, a published case study showed that tralokinumab demonstrated significant efficacy in patients with severe AD accompanied by moderate to mild AA baseline severity AA scores (SALT of 22) (112), thus revealing its potential application in the treatment of comorbidities. Dupilumab, a monoclonal antibody targeting IL-4/IL-13 receptor signaling, has demonstrated significant efficacy in AD and AA, particularly in patients with an atopic backgrounds and elevated IgE levels (113–116). However, its therapeutic response in AA patients presents complexity. On the one hand, dupilumab is effective in improving symptoms in some AA patients; on the other hand, it has been reported that the drug may lead to worsening or new onset of AA (117). This two-sided response may be related to the immune skewed state of patients. In patients with Th2-skewed AA, dupilumab may bring positive efficacy through its inhibitory effect. Although dupilumab may ameliorate symptoms in AA patients with a Th2-skewed immune profile, it can paradoxically exacerbate hair loss in those with Th1-dominant AA, particularly among patients with low IgE levels (118–120). This differential efficacy stems from dupilumab’s dual immunomodulatory effects mediated through IL-4Rα blockade. In comorbid AD/AA characterized by Th2 skewing, dupilumab inhibits Th2 responses, effectively counteracting Th2-driven IgE elevation and barrier damage in AD, while also suppressing Th2 cell-mediated assistance to pathogenic CD8^+^ T cells in AA. Conversely, in Th1-skewed AA lacking significant Th2 activity (e.g., low IgE), dupilumab’s suppression of the Th2 pathway lifts the brake on the key negative feedback control normally exerted over the Th1/IFN-γ axis. This results in excessive amplification of IFN-γ signaling, which in turn promotes CD8^+^ T cell-mediated autoimmune attack on hair follicles, ultimately manifesting as worsened alopecia. In addition, gender differences also affect the efficacy of dupilumab in patients with AA. Studies have shown that female patients are more inclined to Th2 skewed disease, whereas males are more often associated with Th1 skewed disease. Thus, during treatment, female patients (Th2 skewed) are more likely to benefit from dupliyumab therapy, whereas male patients (Th1 skewed) may be at higher risk of deterioration (118). Stratified treatment strategy based on Th1/Th2 skewed status and IgE levels: preferred dopplerizumab for Th2 skewed (high IgE) patients; combined with JAK inhibitors or OX40L antagonists for Th1 skewed (low IgE) patients. Notably, although the prognosis of patients with dupilumab-induced AA worsening is usually favorable, and some patients even remit spontaneously, early recognition and intervention of AA worsening symptoms are crucial for timely adjustment of treatment regimens. Therefore, in clinical practice, physicians should fully consider patients’ immune skewed status and gender differences in order to develop individualized treatment plans and closely monitor changes in disease.

### 6.2 JAK inhibitors

**Figure 4:**
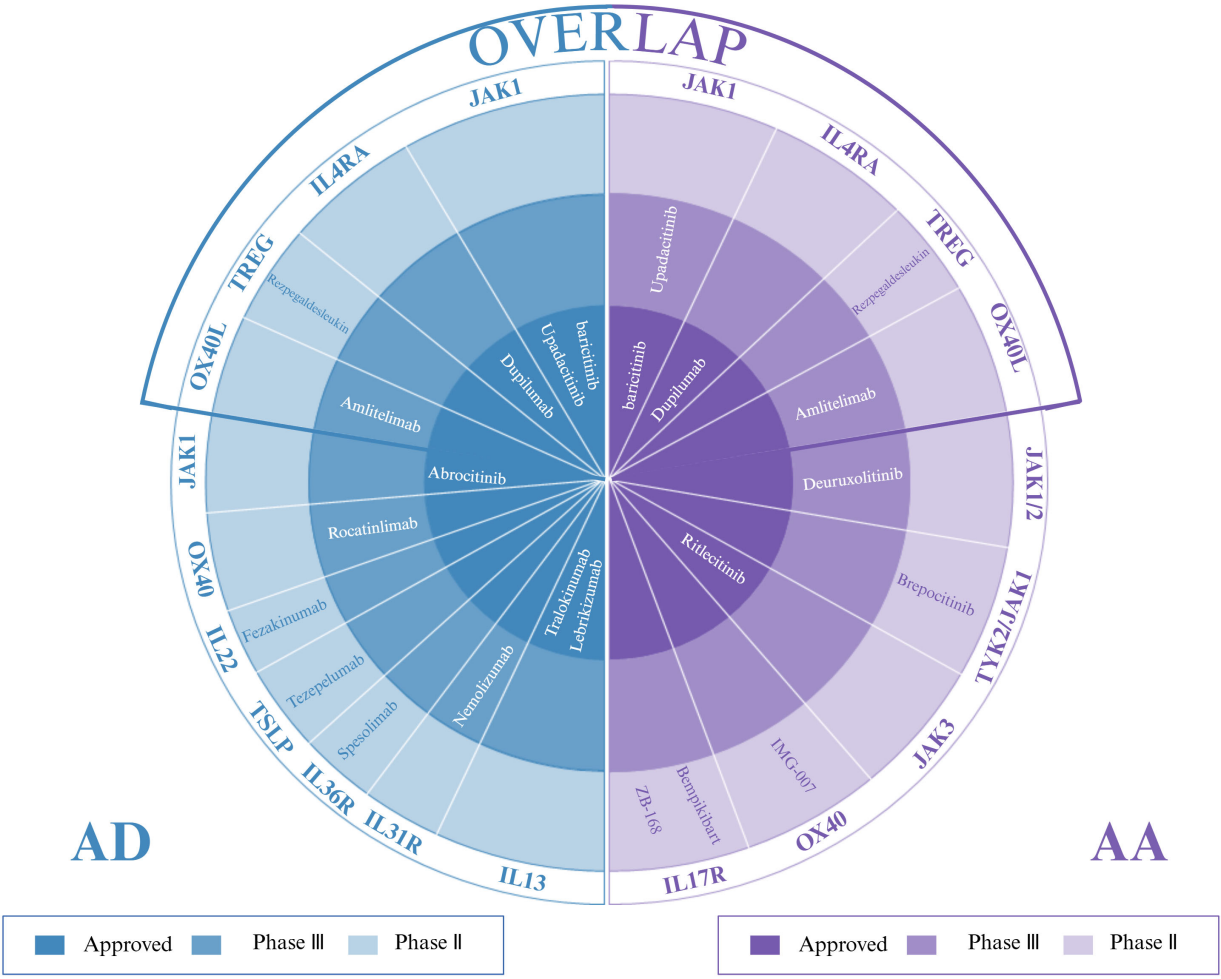
Targeted therapies for AD, AA, and their overlap: approved agents and investigational drugs in phase II/III trials. (Figure created with BioRender.com). AD, atopic dermatitis; AA, alopecia areata.

The JAK-STAT pathway is a key signaling pathway in the development of AD and AA, and JAK inhibitors have shown promising therapeutic efficacy by blocking the signaling of key cytokines in this pathway and suppressing inflammatory responses (121, 122). Compared with first-generation JAK inhibitors (e.g., ruxolitinib and baricitinib), second-generation JAK inhibitors (e.g., upadacitinib and abrocitinib) have demonstrated a significant increase in therapeutic efficacy and have been noted for their higher selectivity and excellent safety profile. In particular, upadacitinib has shown significant efficacy and favorable tolerability in managing comorbid AA and AD comorbidities (123, 124), and its therapeutic potential is particularly promising in AD patients who have failed to respond to or experienced side effects from dupilumab (125). In clinical practice, upadacitinib has not only brought new therapeutic hope to adolescent AA patients with mild AD, but also enabled certain patients with moderate-to-severe AD comorbid with AA, who demonstrated response to baricitinib but discontinued the drug due to side effects, to achieve skin lesion regression and scalp hair regrowth. Real-world evidence documents upadacitinib’s dual efficacy: adolescent AA-AD patients (12–17 years) achieved 80% hair regrowth by 6 months (Child-SALT ≤20), while baricitinib-intolerant adults showed 2.3-fold greater body surface area(BSA) improvement versus baseline (126, 127). Similarly, upadacitinib has shown positive therapeutic effects in adolescent cases of severe refractory AD combined with AA that failed to respond to treatment with dupilumab, as well as in AD patients with refractory AA (128, 129). Despite reports that AA may be induced after upadacitinib treatment for AD (130), the exact mechanism of this complication is not yet clear, and although the efficacy of JAK inhibitors as novel oral small molecule drugs, including baricitinib, upatinib, and abrutinib, is significant, the non-specificity of the mechanism of action raises additional safety consideration (131, 132). In summary, Overall, these findings imply that, as with AD, distinct clinical phenotypes and linked endotypes may characterize patients with AA. Emerging evidence demonstrates the therapeutic promise of second-generation JAK inhibitors (e.g., deuruxolitinib, brepocitinib) in AA-AD comorbidities, yet critical gaps persist in delineating their tissue-specific JAK-STAT pathway modulation and optimal dosing schedules, requiring validation through multicenter Phase IIIb trials coupled with longitudinal biomarker profiling (**Figure 4**).

### 6.3 Other biological agents

Rezpegaldesleukin, a novel biotherapeutic drug in clinical trials, works by specifically targeting the IL-2R complex to stimulate the proliferation of Tregs, thereby inhibiting the aberrant activation of pathogenic T-cell subsets and cytokine storm-mediated excessive immune responses. The drug is currently advancing into clinical studies in several immune-related disease areas and is currently in pivotal phase 2b trials for moderately severe AD and severe to very severe AA (133, 134). These studies will systematically evaluate the therapeutic potential of drugs in different immune disorder scenarios and provide evidence-based medical support for subsequent indication expansion. However, there are still challenges in the field of immunotherapy where efficacy has not met expectations, for example, a phase II study showed that for the treatment of AD, strategies targeting IL-17A in isolation had limited efficacy even in the hyper-activated state of Th17 (135), and that there was no significant difference in the treatment of AD for the anti-IL-22 monoclonal antibody compared to placebo (136). Similarly, no significant efficacy was observed for the anti-IL-17A drug suxinumab in patients with AA (137). In contrast, a variety of drugs targeting the OX40L/OX40 pathway are in phase II/III clinical development for AD treatment and show promise. For example, rocatilinimab has shown good efficacy at all four doses evaluated in its phase 2b trial, with sustained improvement up to 36 weeks post-treatment and efficacy maintained for 5 months after discontinuation. In addition, amlitelimab, by combining with OX40L and blocking its interaction with OX40, has shown rapid improvement in patients with moderate-to-severe AD, with a favorable safety profile, and efficacy was maintained for 6 months after discontinuation, both of which have demonstrated promise for AD treatment. Both demonstrate durable improvement in AD (138). OX40/OX40L inhibitors demonstrate compelling efficacy and favorable safety in AD treatment. Targeting this pathway concurrently depletes multiple pathogenic T-cell subsets (including Th1, Th2, Th17, Th22) and their memory counterparts. Given that aberrant interactions between OX40^+^ T cells and OX40L^+^ antigen-presenting cells (APC) also drive AA progression—a systemic inflammatory disease that may benefit from systemic OX40 inhibition regardless of atopic status—this approach holds therapeutic promise for AA. Although OX40L/OX40-targeted research in AA remains nascent, its potential warrants rigorous clinical validation. In light of these findings, future multicenter cohort studies are needed to clarify biomarkers of AD and AA comorbidity (e.g., OX40L serum levels, FLG mutation status) and to explore the synergistic effects and long-term safety of combination targeted therapies (e.g., JAK inhibitors ^+^ IL-4/IL-13 blockers) (**Table 1**).

**Table 1:** Systematic comparison of the immunopathological mechanisms and treatment responses of AD and AA. Modified from (116, 121, 122).

Comparative Parameters	AD	AA
Immune response patterns	Th2-type immune response, characterized by impaired epidermal barrier function	Th1-type immune response, characterized by disruption of follicular immune privilege
T cell infiltration	Acute phase:Th2 Chronic phase:Th17, Th22, Th1	Core:TH1 Auxiliary:Th2, Th17,Th22
Core cytokines	IL-4, IL-5, IL-13, IL-31, TSLP	IFN-γ, TNF-α, IL-2,IL-17, IL-21
Microbiome characteristics	Skin:Staphylococcus aureus↑ Gut:SCFA↓	Skin:Propionibacterium acnes↑ Gut:SCFA↓
Overlapping therapeutic agents		
Dupilumab (anti-IL-4)	Patients receiving 300 mg of dupilumab every two weeks achieved IGA clearance or near clearance (IGA 0/1) at 16 weeks, significantly higher than the placebo group (36-38% vs. 8-10%)	After 48 weeks of dupilumab treatment, 32.5%, 22.5% and 15% of patients achieved SALT30/SALT50/SALT75 improvement
JAK inhibitors	1. Baricitinib (BREEZE-AD1 trial):In adult patients with moderate to severe AD, the EASI-75 response rate was 24.8% in the 4 mg group, 18.7% in the 2 mg group, and 8.8% in the placebo group 2. Abrocitinib (JADE MONO-1 Trial):In patients aged >12 years with moderate to severe AD, the EASI-75 response rate at week 12 was 63% in the 200 mg group, 40% in the 100 mg group, and 12% in the placebo group 3. Upadacitinib (Measure Up 1 trial):Patients aged >12 years with moderate to severe AD, 79.7% EASI-75 response rate at week 16 in the 30mg group, 69.6% in the 15mg group, and 16.3% in the placebo group	1.Baricitinib:In the BRAVE-AA1 trial (N = 465), the percentage of patients achieving a SALT score ≤20 at week 52 was 40.9% and 21.2% in the 4mg and 2mg baricitinib groups, respectively; In the BRAVE-AA2 trial (N = 390), the percentages were 36.8% and 24.4%, respectively 2. Abrocitinib:Among patients treated with oral abrocitinib (50–200 mg/day) for at least 3 months, 46.15%, 53.85%, and 38.46% achieved a SALT score ≤20, 50% hair regrowth, and 75% hair regrowth, respectively 3. Upadacitinib:In 25 patients (15–30 mg qid), the median absolute SALT score decreased from 50 to 25 at week 12 and further to 5 at week 24
Anti-OX40/OX40L	1. Rocatinlimab: At week 16, the group receiving subcutaneous injections of 300 mg every two weeks showed a significantly greater reduction in EASI scores, at -61.1% (95% CI, -75.2% to -47.0% 2. Amlitelimab: At week 16, the average EASI score decreased significantly in the group receiving 100 mg every 4 weeks, with a reduction of -80.12% (95% CI, -95.55% to -54.60%)	1. IMG-007 demonstrated dose-dependent sustained efficacy in a Phase 2a trial in patients with severe alopecia areata. Patients in the high-dose group experienced an average reduction of 14.3% in SALT at 24 weeks, further decreasing to 21.7% at 36 weeks (NCT06060977) 2. Amlitelimab is currently in Phase 2 clinical trials, with no data yet available (NCTO6444451)
Other	1. Tralokinumab (anti-IL-13):At 16 weeks, tralokinumab 300mg every two weeks was superior to placebo in achieving IGA 0/1 (15.8% vs. 7.1% and 22.2% vs. 10.9%, respectively) and EASI-75 (25.0% vs. 12.7% and 33.2% vs. 11.4%, respectively). 2. Lebrikizumab (anti-IL-13):Lebrikizumab 250mg every two weeks demonstrated efficacy at 16 weeks, achieving IGA 0/1 (33–43%) and EASI-75 (51%−59%) compared to placebo (11–13% and 16–18%, respectively)	1. Tofacitinib (JAK inhibitor): 58% of patients (5–10 mg bid) achieved a SALT score improvement of >50% during 4–18 months of oral treatment 2. Ruxolitinib (JAK inhibitor): Among 12 patients with moderate to severe alopecia areata, 9 achieved an average hair regrowth rate of 92% after 3–6 months of treatment

Th, T helper cells; AD, Atopic Dermatitis; AA, Alopecia Areata; IL, Interleukin; IFN-γ, Interferon-gamma; TNF-α, Tumor Necrosis Factor-alpha; SCFA, Short-Chain Fatty Acids; OX40, Tumor Necrosis Factor Receptor Superfamily Member 4; OX40L, Tumor Necrosis Factor Superfamily Member 4 Ligand; JAK, Janus kinase; TSLP, Thymic Stromal Lymphopoietin; IGA, Investigator’s Global Assessment; SALT, Severity of Alopecia Tool; EASI, Eczema Area and Severity Index; CI, Confidence Interval.

## 7 Probiotic therapy

A study by Enomoto et al. (139) showed that prenatal and six-month postnatal administration of Bifidobacterium shortum M-16V and Bifidobacterium longum BB536 to mothers in combination with their newborns significantly reduced the risk of AD in infants during the first 18 months of life. In addition, specific strains such as Lactobacillus paracasei KBL382 and Lactobacillus sinensis CAU 28(T) showed potential in alleviating AD symptoms and modulating the structure of gut flora (140, 141). Iemoli et al. demonstrated that probiotics balance Th1/Th2 immunity and enhance Treg activity via interactions with dendritic cells (142). However, a randomized controlled trial by Allen et al. found no significant reduction in AD incidence among 2-year-olds receiving probiotic supplementation compared to placebo (143). Subsequent systematic reviews confirm considerable heterogeneity in probiotic efficacy across pediatric, adult, and prenatal populations with AD (144). Although considered a potential therapeutic option, the clinical value of probiotics in AD remains inconclusive with conflicting evidence (145). In a recent randomized, double-blind, placebo-controlled clinical trial, Liu et al. found that FMT therapy could effectively improve the Eczema Area and Severity Index (EASI) scores of patients with AD by regulating the Th2/Th17 ratio, serum TNF-α, and total IgE levels, with good safety, and may serve as a new therapeutic approach for inflammatory diseases (146). Similarly, the long-term hair growth cases observed after FMT provide additional support for the involvement of the gut microbiota in the pathogenesis of alopecia areata (147), but such studies have small sample sizes and lack controls. Based on the limited studies identified in the literature, a clear association between gut microbiota dysbiosis and alopecia areata has not yet been established. It is worth noting that AA is strongly associated with nutritional factors, especially vitamin D deficiency and its receptor low expression are associated with AA (148, 149). Given that vitamin D receptor expression is regulated by the gut microbiota (150) and that the gut microbiome influences nutrient absorption, it has been hypothesized that reversal of gut dysbiosis may improve the absorption of hair-growth-friendly nutrients such as vitamin D (151). Recent studies have shown that exploratory therapies such as fecal microbial transplantation (FMT) exhibit potential in reducing AD severity (152), although these findings are largely based on small experimental studies. Similarly, cases of long-term hair growth observed after FMT provide additional support for the involvement of the gut microbiome in AA pathogenesis (153), although the statistical significance of the results of tests for changes in gut flora in some AA patients was not significant (154).

## 8 Conclusions and perspectives

This review has systematically examined the intricate relationship between AD and AA, focusing on their overlapping epidemiological links, shared pathophysiological mechanisms, and implications for therapeutic strategies. We first delineated the robust bidirectional epidemiological association between these conditions, highlighting AD as a significant risk factor for AA development and vice versa. Subsequently, we dissected the complex immune networks underpinning both diseases, moving beyond the traditional Th1/Th2 dichotomy to emphasize the concurrent dysregulation and crosstalk among multiple axes, including Th1 (IFN-γ, TNF-α), Th2 (IL-4, IL-13, IgE), Th17 (IL-17), and Th22 (IL-22). Key signaling pathways, notably JAK-STAT and OX40-OX40L, were identified as critical convergent hubs driving inflammation, barrier dysfunction, and follicular damage in both AD and AA. Shared genetic susceptibility loci, particularly involving the FLG gene and Th2 cytokines (IL-4, IL-13), further solidify their common etiological framework. The review also explored the emerging role of gut and skin microbiome dysbiosis, characterized by reduced SCFA production and increased intestinal permeability (“leaky gut”), in modulating systemic and local immune responses that exacerbate both conditions.

Collectively, the convergence of genetic predisposition, immune dysregulation across multiple axes, and microbiome-mediated modulation creates a shared pathophysiological landscape for AD and AA. We propose that the active inflammatory state in one disease may predispose to or exacerbate the other, reflecting their mechanistic interplay. Critically, this mechanistic overlap holds significant therapeutic implications. Emerging evidence suggests that targeted therapies effective for one condition may exert beneficial effects on the other, as exemplified by the potential efficacy of certain IL-4/IL-13 inhibitors and JAK inhibitors in comorbid presentations (**Figure 3**). However, the bidirectional response observed with agents like dupilumab underscores the complexity and the need for patient stratification based on immune endotypes (e.g., Th1 vs. Th2 skew, IgE levels).

Despite these advances, critical knowledge gaps persist. The precise pathophysiological interplay mediated by the microbiome in the AD-AA dyad requires further elucidation through integrated multi-omics approaches. The long-term efficacy and safety profiles of novel biologics targeting pathways like OX40-OX40L in AA, either alone or in combination with existing agents (e.g., JAK inhibitors), warrant rigorous investigation in large-scale clinical trials. Furthermore, reliable biomarkers predictive of comorbid risk, disease severity, and therapeutic response (e.g., serum OX40L levels, specific microbial signatures, *FLG* mutation status) remain to be robustly defined. Future research should prioritize elucidating these unresolved questions and optimizing personalized therapeutic strategies, including exploring synergistic combinations of targeted agents and microbiota-modulating interventions (e.g., defined probiotics, FMT), to effectively manage the challenging clinical scenario of overlapping AD and AA.
